# Targeting RNA-binding protein HuR to inhibit the progression of renal tubular fibrosis

**DOI:** 10.1186/s12967-023-04298-x

**Published:** 2023-06-30

**Authors:** Zhimin Huang, Simeng Liu, Anna Tang, Xiaoqing Wu, Jeffrey Aube, Liang Xu, Yufeng Huang

**Affiliations:** 1 Division of Nephrology & Hypertension, Department of Internal Medicine, University of Utah Health Science, Wintrobe Rm 403, 26 N Medical Dr., Salt Lake City, UT 84132 USA; 2 Department of Molecular Biosciences, University of Kansas, Lawrence, KS USA; 3 Department of Chemical Biology and Medical Chemistry, Eshelman School of Pharmacy, University of North Carolina, Chapel Hill, NC USA

**Keywords:** RNA-binding protein, HuR inhibitor, Ischemia–reperfusion, Renal fibrosis, Chronic kidney disease

## Abstract

**Background:**

Upregulation of an RNA-binding protein HuR has been implicated in glomerular diseases. Herein, we evaluated whether it is involved in renal tubular fibrosis.

**Methods:**

HuR was firstly examined in human kidney biopsy tissue with tubular disease. Second, its expression and the effect of HuR inhibition with KH3 on tubular injury were further assessed in a mouse model induced by a unilateral renal ischemia/reperfusion (IR). KH3 (50 mg kg^−1^) was given daily via intraperitoneal injection from day 3 to 14 after IR. Last, one of HuR-targeted pathways was examined in cultured proximal tubular cells.

**Results:**

HuR significantly increases at the site of tubular injury both in progressive CKD in patients and in IR-injured kidneys in mice, accompanied by upregulation of HuR targets that are involved in inflammation, profibrotic cytokines, oxidative stress, proliferation, apoptosis, tubular EMT process, matrix remodeling and fibrosis in renal tubulointerstitial fibrosis. KH3 treatment reduces the IR-induced tubular injury and fibrosis, accompanied by the remarkable amelioration in those involved pathways. A panel of mRNA array further revealed that 519 molecules in mouse kidney following IR injury changed their expression and 71.3% of them that are involved in 50 profibrotic pathways, were ameliorated when treated with KH3. In vitro, TGFβ1 induced tubular HuR cytoplasmic translocation and subsequent tubular EMT, which were abrogated by KH3 administration in cultured HK-2 cells.

**Conclusions:**

These results suggest that excessive upregulation of HuR contributes to renal tubulointerstitial fibrosis by dysregulating genes involved in multiple profibrotic pathways and activating the TGFß1/HuR feedback circuit in tubular cells. Inhibition of HuR may have therapeutic potential for renal tubular fibrosis.

**Supplementary Information:**

The online version contains supplementary material available at 10.1186/s12967-023-04298-x.

## Background

Renal fibrosis, particularly tubulointerstitial fibrosis, is the hallmark and the final common pathway of all progressive chronic kidney diseases (CKD) ultimately leading to end-stage renal disease (ESRD) [1, 2]. Tubulointerstitial fibrosis consists of many major events. Uncontrolled and persistent inflammation after the initial insults of various injuries induces renal fibrogenic response and activates and expands the matrix-producing cells from various sources through multiple mechanisms, including activation of interstitial fibroblasts and pericytes and activation of epithelial-to-mesenchymal transition (EMT) of tubular cells [1, 3]. Current therapies indirectly for renal fibrosis mostly focus on regulating the renin–angiotensin system including angiotensin-converting enzyme inhibitors and angiotensin receptor blockers, and only slow CKD progression [4]. Other therapies have also provided moderate results, such as the endothelin-1 receptor antagonist, atrasentan or the sodium–glucose transporter 2 inhibitor [5]. Unfortunately, though, no effective and direct strategies exist to block or reverse CKD and prevent ESRD.

Human antigen R (HuR), known as embryonic lethal abnormal vision-like 1 (ELAVL1), is a ubiquitously expressed RNA binding protein [6]. Normally, HuR is mainly localized within the nucleus of resting cells. Under various forms of abnormal stimuli, HuR binds to adenine and uridine rich sequence elements (AREs) in mRNA 3′-untranslated region (3′-UTR), transports mRNA to cytoplasm, and protects mRNA from rapid degradation, and thereby enhancing those targets’ generation and action [7]. It has been shown that HuR regulates mRNA turnover and translation of a variety of genes involved in immune response, inflammation, angiogenesis, EMT, fibrosis, and oncogenic signaling pathways in several types of cancer [8–11]. The role of HuR in organ fibrosis has attracted increasing attention since all above enhanced events occurred in cancer are also mainly involved in the development and progression of organ fibrosis. In fact, it has been observed that cytoplasmic HuR expression was elevated in renal glomerular epithelial cells, mesangial cells and tubular cells in human and rodent diabetic and nondiabetic nephropathy specimens along with abnormal proliferation, inflammation, EMT changes compared to normal controls [12–17]. Therefore, selective inhibition of HuR might provide us a promising way to simultaneously and directly inhibit multiple profibrotic pathways involved in the development and progression of CKD.

Recently, we have discovered a series of small molecule HuR inhibitors at nM to sub-µM Ki values, which dose-dependently inhibit the action of HuR by specially disrupting HuR-ARE interaction [18, 19]. The lead compound KH3 (MW, 430.54) exhibits superior potency in disrupting HuR-ARE with a Ki of 0.7 µM. Of note, the MTT-based cytotoxicity of KH3 in a panel of cell lines revealed that KH3 at optimal dose selectively inhibited the viability of cells such as cancer cells that had high levels of HuR (IC50 < 10 µM), but had no effect on normal cell line that had low HuR (IC50 = 46 µM) [19]. This unique feature of KH3 that may not affect normal cells may place it as an ideal candidate of HuR inhibitor for future clinical application. Importantly, therapeutic effect of KH3 has been observed in ameliorating pathological cardiac hypertrophy and fibrosis and renal proteinuria and glomerulosclerosis and preserving cardiac and renal function in animal models [17, 20]. However, little is known about HuR expression and the effect of HuR inhibition in the progression of renal tubulointerstitial fibrosis. In this study, HuR was first examined in human kidney biopsy tissues. In addition, renal warm ischemia/reperfusion (IR) model in mice creates severe tubular injury, interstitial infiltration of inflammatory cells and renal fibrosis, which presents with many similarities to the pathophysiology of progressive renal tubulointerstitial disease in patients [21]. Furthermore, it has been widely recognized that ischemic renal failure is an important risk factor leading to the occurrence and progression of CKD [22]. Therefore, IR-induced kidney disease model was used in the present study to examine HuR expression and the therapeutic potential of HuR inhibition in the pathophysiologic pathways that focus on renal tubular injury and fibrosis. Furthermore, the mechanisms of anti-fibrotic action of HuR inhibition involved were further analyzed both in vivo and in vitro in cultured renal tubular epithelia cells in the present study. Our studies further observed upregulation of HuR in tubular and tubulointerstitial cells in both CKD patients and mouse models. Inhibition of RNA-binding protein HuR protected kidney from IR-induced injury and subsequent tubulointerstitial fibrosis via inhibiting inflammatory response and the majority of fibrotic pathways, suggesting that HuR-targeted inhibitory therapeutics offer a promising novel treatment for preventing or reversing the progression of CKD.

## Methods

### Reagents

The HuR inhibitor KH3 was synthesized as previously described [18, 19]. KH3 powder was dissolved in DMSO at 20 mM as stock solutions for in vitro assays, or in PBS with 5% ethanol and 5% Tween-80 for in vivo animal studies [19]. Unless otherwise stated, all other reagents were purchased from Sigma-Aldrich Chemical Co. (St. Louis, MO, USA).

### Study 1. In situ changes of RNA-binding protein HuR in progressive CKD patients

To observe HuR expression in CKD patients with tubular injury and fibrosis, human renal tissue was obtained for tubular HuR staining from archived paraffin-embedded biopsy material that we used for glomerular HuR staining previously [17]. Fifteen initial renal biopsy sections were obtained from CKD patients with advanced mesangial proliferative IgA nephropathy (IgAN, n = 3), or diabetic nephropathy (DN, n = 3), or lupus nephritis (LN, n = 3), or focal segmental glomerulosclerosis (FSGS, n = 3), or thrombotic microangiopathy (TMA, n = 3) before receiving any treatment but developed renal tubulointerstitial fibrosis. Three or four-µm-thick renal sections were stained for PAS and HuR described later and carried out as described in Study 2. The tubular HuR levels in CKD patients besides the enhanced glomerular HuR levels determined previously were assessed by the relative integrated pixel density of the staining of HuR specially in renal tubulointerstitial area determined by Image-J (National Institutes of Health, Bethesda, Maryland, USA), and compared with normal kidney sections (NC, n = 3) that were obtained from three different normal part of surgically removed tumor kidneys. Use of human material was reviewed and approved by the Institutional Review Board (IRB) of the University of Utah.

### Study 2. In vivo studies of changes and inhibition of RNA-binding protein HuR in ischemia–reperfusion-induced kidney injury and fibrosis in a mouse model

#### Animals

Male mice (C57BL/6) at age of 10 weeks were obtained from the Jackson Laboratory (Bar Harbor, ME, USA), housed in standard cages with a 12 h light/dark cycle and given water and normal diet ad libitum. Animal maintenance and study procedures described herein were carried out at University of Utah in accordance with Public Health Service Policy on Use of Laboratory Animals and approved by the Institutional Animal Care & Use Committee (IACUC) of the University of Utah.

#### Experimental design

All mice (n = 10) at age of 12 weeks were received unilateral renal ischemia–reperfusion (IR) surgery as described previously [23]. Mice were then randomly assigned into either vehicle-treated (n = 5) or KH3-treated group (n = 5). KH3 at doses of 50 mg kg^−1^ B.W.day^−1^ determined previously [17] was given to treated mice daily via intraperitoneal injection, started at 72 h (day 3) after IR surgery when fibrotic reaction starts. Simultaneously, the vehicle treated mice received daily intraperitoneal injection of buffer alone. On day 14 after IR surgery, all mice were euthanized under isoflurane anesthesia. Blood and kidney samples were harvested in the same way as described previously [23].

#### Determination of renal and liver function

Plasma BUN and creatinine (Cr) concentrations were measured by using the QuantiChrom™ urea assay kit and creatinine assay kit (BioAssay System, Hayward, CA, USA). Plasma Alanine aminotransaminase (ALT) levels indicating liver function were detected using the Heska Element DC Chemistry Analyzer (Heska Fuji Film Corporation, Loverland, CL, USA).

#### Histological examination

Three-micrometer sections of paraffin-embedded kidney tissues were stained with periodic acid-Schiff (PAS) and Masson’s Trichrome (TRI) by the histology core facility at University of Utah and scored in a blinded fashion with a semi-quantitative ordinal scale as described previously [23].

Immunofluorescent staining (IF) for HuR was performed on paraffin-embedded kidney tissues as described previously [17]. At the same time, Alexa Fluor™ 488-conjugated wheat germ agglutinin (WGA) (ThermoFisher Scientific, USA) was used to counter-stain the glomeruli and tubules to define the location of HuR-positive cells in the kidney. DAPI-Fluoromount-G (SouthernBiotech, Birmingham, AL, USA) was used to stain the nuclei DNA to indicate cellular position of HuR. Control slides treated with antibody diluent instead of primary antibody of HuR showed no staining.

Immunofluorescent staining for α-smooth muscle action (α-SMA) or CD31+, or F4/80+ or Ki-67+ positive cells were performed and quantified on frozen kidney sections as described previously [23–25]. Either polyclonal rat anti-mouse F4/80 IgG (Bio-Rad Laboratories, Inc., Hercules, CA, USA), or monoclonal rat anti-Ki-67 antibody, (Invitrogen) or rat anti-mouse CD31 IgG2a (BD Biosciences, San Jose, CA, USA) served as the primary antibody respectively. The Cy^TM3^-conjugated goat anti-rat IgG (Jackson ImmunoResearch Laboratories Inc., West Grove, PA, USA) was used as the secondary antibody. Control slides treated with antibody diluent instead of primary antibody showed no staining. For immunostaining of α-SMA, FITC-conjugated mouse anti-α-SMA antibody was used directly. After staining, one drop of DAPI-Fluoromount-G (SouthernBiotech) was applied on the section to counter-stain the nuclei DNA. Ten random fields from each section were analyzed under 200× magnification. Digital morphometric measurement of F4/80+, or Ki-67+, or CD31+-positive cells or α-SMA positive staining was quantified separately using Image-J (National Institutes of Health, Bethesda, Maryland, USA).

#### Western blot measurement

Kidney protein from each animal of each group was isolated, pooled at equal amount to represent the individual group and then immunoblotted on immobilon-P transfer membranes (ThermoFisher Scientific) as described previously [17, 23]. Proteins of HuR, α-SMA, fibronectin (FN), E-cadherin, N-cadherin, vimentin transforming growth factor-ß1 (TGFß1), PAI-1, nuclear factor κ-B p65 unite (NF-κB-p65), NADPH oxidase gp91^phox^ (Nox2) and its cofactor, p47^phox^, Nox4, phosphorylated and total ERK1/2 and GAPDH were assessed on the blots. The antibody information and analysis of the immunostaining bands were described previously [17, 23, 24, 26, 27]. All blots were run at least three times.

The resources and working dilution fold of the primary and secondary antibodies used for immunofluorescent staining and western blot measurement in detail were listed in the Additional file 1: Table S1 (Part I and Part II).

#### RNA isolation and real-time RT-PCR assay

Total RNA from kidney tissue of each mouse was extracted using TRIzol reagent (ThermoFisher Scientific). Real-time RT-PCR was performed using the superscript III first-strand synthesis kit, the power SYBR green PCR master mix (ThermoFisher Scientific) and the ABI 7900 Sequence Detection System (Applied Biosystems) as described previously [17, 23, 24]. Samples were run as triplicates in separate tubes to permit quantification of the target gene normalized to GAPDH and analyzed by 2^(−∆∆Ct)^. The relative mRNA levels of target gene were expressed relative to the untreated contralateral kidney control (CTL), which was set at unity. Sequences of primers used for targeted molecules including mouse and human molecules (as noted later) were listed in Additional file 1: Table S2.

#### In situ cell death detection

A FITC-labelled the enzyme terminal deoxynucleotidyl transferase-mediated dUTP nickend labeling (TUNEL) assay that labels the damage DNA for the detection of apoptosis was carried out in kidney sections in situ as described previously [23]. The apoptotic index for each kidney was calculated as the number of TUNEL-positive cells/100 cells from 10 arbitrarily chosen higher power fields.

#### NanoString mRNA expression assay

A digitalized multiplex NanoString nCounter mouse mRNA expression assay (NanoString Technologies, Inc., USA) was performed with 100 ng of pooled RNA samples isolated from contralateral kidneys or IR-injury kidneys treated with or without KH3 as described previously [23]. Briefly, RNA samples were added to the codeset duplicated and hybridized to probes at 65 °C for 16 h. The codeset comprised of reporter and capture probes that were hybridized with the target mRNA sequences, establishing a tripartite complex. After incubation, the counts for the tripartite complex representing each molecule’s mRNA expression were recorded. The gene expression data was analyzed by bioinformatic core faculty by normalization to the mean of positive control probes and the geometric mean of reference genes.

### Study 3. In vitro studies of the effect of inhibition of RNA-binding protein HuR on TGFß1-induced renal tubular EMT

#### Cell culture

Human renal tubular epithelial cells (HK-2) were originally obtained from the American Type Culture Collection (ATCC, Rockville, MD, USA) and maintained in DMEM/F12 medium supplemented with 10% fetal bovine serum (FBS), 100 μg/mL streptomycin and 100 U/mL penicillin (all from Gibco, Thermo-Fisher Scientific, Waltham, MA, USA) at 37 °C in a 5% CO_2_ incubator. Sub-confluent cells seeded on six-well plates were made quiescent in serum-free DMEM/F12 medium for 24 h before experimental studies. All cellular treatments were carried out at University of Utah, as duplicates in separate wells and repeated for three times.

#### Effect of TGFß1 on cellular HuR expression and cytoplasmic translocation

The quiescent cells were treated with recombinant human TGFβ1 (5 ng/mL, R&D System, USA), and then collected at 0, 3, 6, 12, 24,48 h after treatment for HuR immunocytofluorescent staining as described above. Forty-eight-hour incubation time was then chosen as an optimal time for TGFß1 to induce cytoplasmic translocation of HuR with or without HuR inhibitor, KH3, in cultured HK2. Briefly, the quiescent cells were incubated in serum-free medium alone, or serum-free medium with KH3 (10 μM), or TGFβ1 (5 ng/mL) or TGFβ1 (5 ng/mL) plus KH3 (10 μM). KH3-treated cells were preincubated with KH3 for 30 min before adding TGFβ1. Cells were harvested after 48-h incubation for HuR staining (described above) and measurement of mRNA of HuR and cytoplasmic/nuclear/total cellular HuR protein production by real-time RT-PCR and Western blotting as described above. Differently, cytoplasmic and nuclear protein was isolated separately by using Thermo-Scientific™ NE-PER™ Nuclear and Cytoplasmic Extraction Reagents kit (ThermoFisher Scientific). A rabbit anti-Histone H3 antibody and a goat anti-GAPDH antibody were used to determine the equal loading of nuclear protein and cytoplasmic protein respectively.

#### Effect of KH3 on TGFß1-induced renal tubular EMT

The quiescent cells were incubated in serum-free medium with or without TGFß1(5 ng/mL) in the presence or absence of KH3 (10 μM) for 48-h and then harvested for analysis of mRNA and protein expression of TGFß1, PAI-1, Nox4, FN, a-SMA, E-cadherin and N-cadherin by real-time RT-PCR and Western blotting as described above.

### Statistical analysis

All data are expressed as mean ± SD. Software Power for sample size calculation (www.clincalc.com) was used for in vivo study, based the results of renal tubular injury score in a pilot study. Each group contains 5 mice and the study has at least 95% power to detect differences larger than 2.2 units of standard deviation between treated and untreated groups. Statistical analyses of differences among the groups were performed by two ways-ANOVA and subsequent Student–Newman–Keuls or Dunnett’s testing for multiple comparisons. Comparisons with p values < 0.05 were considered significantly different.

## Results

### Elevated renal tubular HuR is detected in progressive CKD patients

**Fig. 1:**
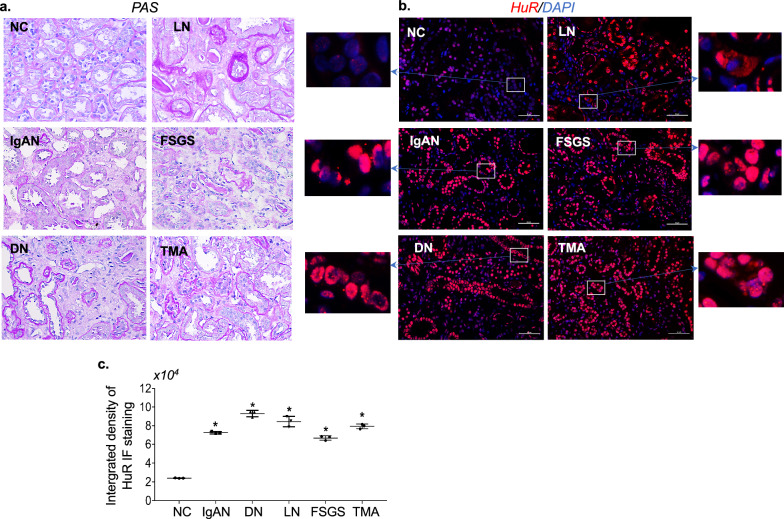
Renal tubulointerstitial HuR expression was elevated in progressive CKD patients. **a**, **b**. Representative microphotographs stained for PAS (**a**) from kidney biopsy tissue of patients with progressive IgA nephropathy (IgAN, n = 3), or diabetic nephropathy (DN, n = 3), or lupus nephritis (LN, n = 3), or focal segmental glomerulosclerosis (FSGS, n = 3), or thrombotic microangiopathy (TMA, n = 3) (×400 magnification) and their immunofluorescent stained for HuR (red) and DAPI (blue) (**b**), compared with normal kidneys (NC, n = 3). Tubular cytoplasm staining for HuR was clearly seen in the enlarged photos. Scale bars for HuR staining photos represent 50 µm. **c** Relative integrated pixel density of the staining of HuR specially in renal tubulointerstitial area, as quantified by image-J. *P < 0.05 vs. normal kidney (NC)

We have taken 15 renal biopsy tissues from CKD patients with different pathological conditions. As per our previous report, glomerular HuR staining has been significantly elevated in all the diseased glomeruli in comparison to normal glomeruli [17]. Here, PAS-stained kidney sections showed a certain extent of tubular injury and interstitial fibrosis (Fig. 1a). Notably, IF staining using anti-HuR antibody revealed that tubulointerstitial HuR (red) was increased and the cytoplasmic shift of HuR in tubular cells was also able to be observed in those patients with advanced either IgAN, or DN, or LN, or FSGS, or TMA (Fig. 1b and enlarged photos with cytoplasm staining for HuR). The staining levels of total tubular HuR in diseased kidneys quantified by Image-J were evidently augmented compared to normal kidneys (Fig. 1c). Further evaluation of HuR staining and analysis in a large number of CKD patients is needed. Nonetheless, our results showed the over-expression of renal HuR which might be correlated as a key feature of progressive CKD in humans.

### Elevated HuR is observed in IR-injured kidneys in a mouse model

**Fig. 2:**
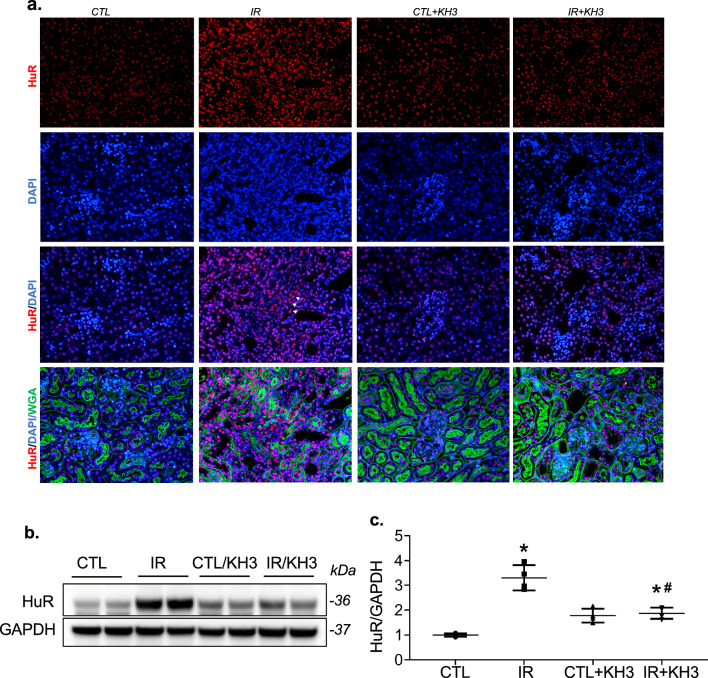
Treatment with KH3 reduces renal tubulointerstitial HuR staining and protein expression in the ischemia–reperfusion (IR)-injured mouse kidney. **a** Representative photomicrographs of renal immunofluorescent staining for HuR (red), DAPI (blue) and wheat germ agglutinin (WGA) (green) (×200 magnification) from the un-injured contralateral kidney (CTL) and IR-injured kidney treated without (IR) or with KH3 (CTL+KH3, IR+KH3). Cytoplasm staining for HuR was able to be seen in IR-injured kidneys as arrows indicated. **b** Representative Western blots illustrated protein expression of HuR and ß-actin in the whole kidney tissue (n = 5/each group). **c** Quantification of the band density is shown on the right of Western blot. Protein values are expressed relative to normal control, which was set at unity. KH3 treatment resulted in a significant reduction in renal tubular and tubulointerstitial HuR staining and protein production levels compared with IR-injured kidneys. *P < 0.05, vs. CTL; ^#^P < 0.05, vs. IR

As shown in Fig. 2a, HuR protein (stained red) was predominantly localized within the nucleus of all tubular cells and its staining intensity was weak in the kidney cells without IR injury (CTL). There was no cytoplasmic staining of HuR was observed in tubular cells of the CTL kidneys. In contrast, HuR staining intensity was markedly increased in the nucleus in tubular and tubulointerstitial cells and the cytoplasmic staining for HuR was able to be observed in many of these cells in the IR-injured mouse kidneys (as arrows indicated). Treatment with KH3, both the number of cells in the tubulointerstitial area and the staining intensity of HuR were reduced compared with those untreated disease controls. No significant changes in IF staining intensity and pattern for HuR were observed in glomerular cells with or without IR injury, which was consistent with the fact that this animal model mainly caused renal tubular injury. Consistent with this, immunoblots detected a significant increase in HuR protein levels in renal protein extracted from IR-injured kidneys, compared with the CTL kidneys, which were partially decreased by treatment with KH3 (Fig. 2b, c). These results indicate that enhanced HuR expression is observed in kidney cells at the site of injury following IR in a mouse model.

### Safety of KH3 administration

There was no difference in daily food intake, water intake and body weight changes between KH3-treated and untreated IR-injured mice. None of mice had diarrhea or peritonitis. The plasma ALT activity in KH3-treated mice was the same as that of mice without treatment, which was less than 10 U/L, indicating no any liver damage. In addition, serum BUN and creatinine (Cr) levels in KH3-treated mice were significantly lower than those in untreated mice, suggesting KH3 treatment may ameliorate the impaired renal function induced by IR injury (BUN, vs KH3 treated, 26.82 ± 4.26 vs 17.53 ± 2.39 mg/dL, P < 0.05; Cr, 0.302 ± 0.03 vs 0.191 ± 0.02 mg/dL, P < 0.05). These results suggest that KH3 given at the proper dose is well-tolerated with no major safety concerns in the present mouse model, as we observed in nephritic rats [17].

### Treatment with KH3 protects kidney from IR-induced injury and fibrosis in mice

**Fig. 3:**
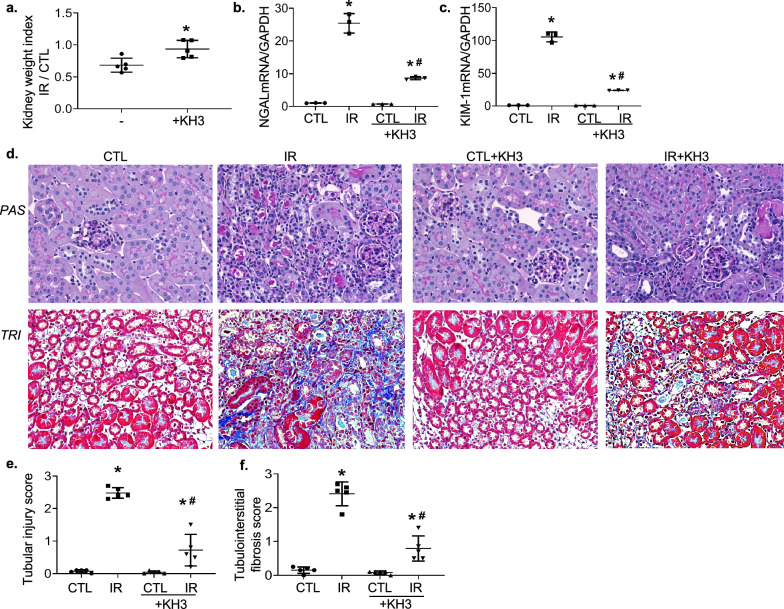
Treatment with KH3 ameliorates mouse renal atrophy and IR-induced tubular injury and fibrosis. **a**–**c** kidney weight (**a**), mRNA expression of tubular injury markers, NGAL (**b**) and KIM-1 (**c**) (n = 5/group). **d** Representative microscopic images showing PAS staining and Masson’s Trichrome staining of the kidney sections used to detect tubular injury, inflammation and collagen deposition (stained blue). Magnification, ×200. **e**–**f** The graphs summarized the results of tubular injury (**e**) and collagen deposition (**f**) quantified by image-J. *P < 0.05 vs untreated mice or contralateral kidney (CTL). ^#^P < 0.05 vs untreated IR-injured kidney (IR)

The IR-injured kidney in mice showed kidney atrophy as evident by decreased kidney weight index (ratio of injured-kidney weight over control, uninjured-kidney weight) (Fig. 3a), with markedly elevated tubular injury markers such as sustained overexpression of neutrophil gelatinase-associated lipocalin (NGAL) and kidney injury molecule-1 (KIM-1) mRNA (Fig. 3b, c). The latter is usually correlated with the extent of renal tubular inflammation and fibrosis. In addition, the IR-injured kidney showed extensive tubulointerstitial injury, which was characterized by structurally disordered, necrotic renal tubular epithelial cells, detached brush border, various tubular casts and massive infiltration of inflammatory cells, disappeared physical back to back position with enlarged spaces in between, and accumulated deposition of collagen as determined by PAS staining and Masson’s trichrome staining (Fig. 3d–f). In contrast, treatment with KH3 resulted in a significant reduction in the NAGL and KIM-1 expression levels in the IR-injured kidneys compared to untreated injured kidneys, accompanied by drastically reduced kidney atrophy, tubular injury, and collagen deposition. On the other hand, treatment with KH3 had no effect on normal kidneys. These results indicate that tubular damage and fibrosis following IR injury are reduced in KH3-treated mice.

**Fig. 4:**
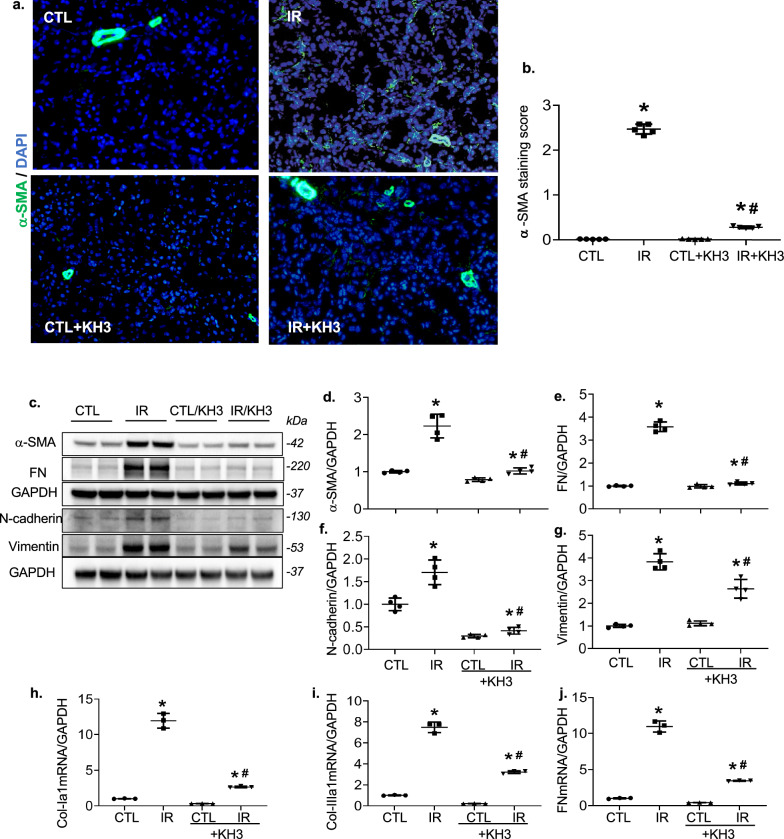
Treatment with KH3 reduces tubular a-SMA staining and renal protein levels of fibrotic markers following ischemia–reperfusion (IR) injury. **a** OCT kidney sections from mice following IR injury without and with KH3 treatment were stained with a-SMA (green) and DAPI/DNA (blue) and analyzed using confocal microscopy. Magnification, ×200. **b** The graphs summarized the results of a-SMA deposition quantified by image-J. **c** Western blots of a-SMA, FN, N-cadherin, vimentin and GAPDH from CTL and IR-injured kidneys of KH3-untreated and treated mice. Molecular weight was labelled on the right. N = 5 animals/group. **d**–**g** The graphs summarize the results of band density measurements for a-SMA (**d**), FN (**e**), N-cadherin (**f**) and Vimentin (**g**). **h**–**j** mRNA expression of Col-I (**h**), Col-III (**i**) and FN (**j**) in the kidney following IR injury. *P < 0.05 vs. untreated contralateral kidney (CTL); ^#^P < 0.05 vs. untreated IR-injured kidney (IR)

The immunofluorescent stained kidney sections for α-SMA and its semi-quantitative analysis have been shown in Fig. 4a, b. The IR-injured kidney sections showed over-expression of α-SMA around renal tubular cells, in addition to its staining normally in the vessels, as compared to the uninjured CTL kidneys. However, KH3 treatment markedly suppressed the α-SMA expression. This observation was further supported by Western-blot data revealing an increased protein level of α-SMA in IR-injured kidneys; along with increased renal protein levels of FN, N-cadherin, and vimentin which are considered as mesenchymal/fibrotic phenotypic markers (Fig. 4c–g). KH3 treatment significantly ameliorated these protein levels (Fig. 4c–g). Consistently, elevated mRNA expression of collagen type Iα1 (Col-Iα1), Col-IIIα1, and FN, as fibrotic markers, determined by real time RT-PCR assay, was further observed in the untreated and IR-injured kidney, which was markedly reduced by KH3 treatment (Fig. 4h–j). Together, these results revealed that KH3 treatment suggestively protects renal tissue against IR-induced tubular EMT and fibrosis.

### Treatment with KH3 attenuates HuR-targeted molecules that are critical for renal inflammatory and fibrotic responses following IR injury in mice

**Fig. 5:**
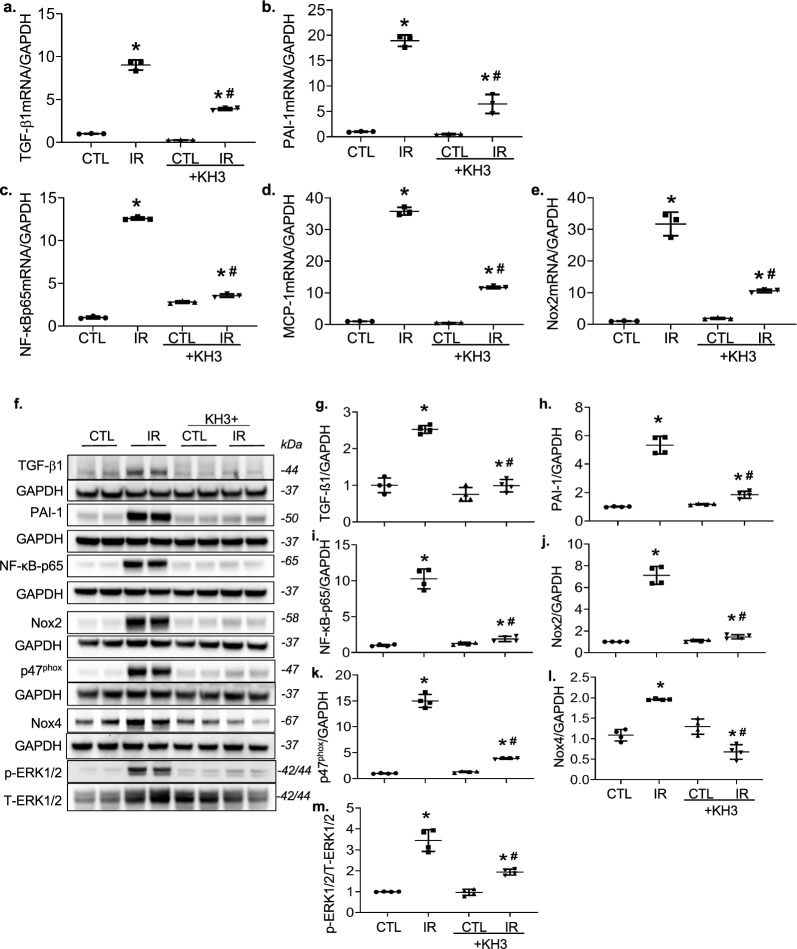
Treatment with KH3 reduces renal mRNA expression and protein production of profibrotic, pro-inflammatory and proliferative markers in the ischemia–reperfusion (IR)-injured mouse kidney. **a**–**e**, Expression of TGFß1 (**a**), PAI-1 (**b**), NF-kBp65 (**c**), MCP-1 (**d**), and Nox2 (**e)** mRNA was determined by real-time RT/PCR. Changes in mRNA levels were determined by first correcting the amplification of GAPDH for each sample. **f** Representative Western blots of TGFß1, PAI-1, NF-kBp65, Nox2, p47phox, Nox4 and GAPDH and pERK1/2, t-ERK1/2 from contralateral (CTL) and ischemia–reperfusion (IR)-injured kidneys of untreated and KH3-treated mice. **g**–**m** The graphs summarize the results of band density measurements for TGFß1 (**g**), PAI-1 (**h**), NF-kBp65 (**i**), Nox2 (**j**), p47phox (**k**), Nox4 (**l**) and pERK1/2/t-ERK1/2 (**m**), respectively. *P < 0.05 vs. untreated contralateral kidney (CTL); ^#^P < 0.05 vs. untreated IR-injured kidney (IR). n = 5/each group

Both of TGFß1 and PAI-1, two key pro-fibrotic modulators, have been identified as the HuR targets [14, 28]. As expected, renal mRNA levels of TGFβ1 and PAI-1 were increased in IR-injured kidneys, compared with their internal CTL un-injured kidneys (Fig. 5a, b). Consistently, renal protein production of TGFß1 and PAI-1 was much greater in IR-injured kidneys (Fig. 5f–h). However, both mRNA and protein levels of these two molecules were reduced in IR-injured kidneys treated with KH3.

It has been shown that most proinflammatory transcripts comprehend conserved or semiconserved AREs in their 3’UTR of mRNA molecules [29]. Thus, enhanced HuR may further upraise inflammatory mediators seen in IR-injured kidney cells. We then examined renal mRNA levels of NADPH oxidase-2 (Nox2), NF-κB-p65 and monocyte chemoattractant protein-1 (MCP-1) in the kidney tissues of IR-injured mice. We found that the above-mentioned markers were over-expressed in IR-injured kidneys (Fig. 5c–e). Additionally, we found the elevated renal protein levels of Nox2, its co-factor p47^phox^, NF-κB-p65 and Nox4 in IR-injured kidneys in comparison to CTL kidneys, signifying substantial activation of NF-κB-mediated inflammatory pathway and NADPH oxidases within the IR-injured kidney tissue. Of note, KH3 treatment significantly decreased those mediators (Fig. 5i–l). Moreover, renal phosphorylation of ERK1/2 was found to be increased by IR-injury, which was reduced, approaching normal levels, in KH3-treated mice (Fig. 5f, m).

**Fig. 6:**
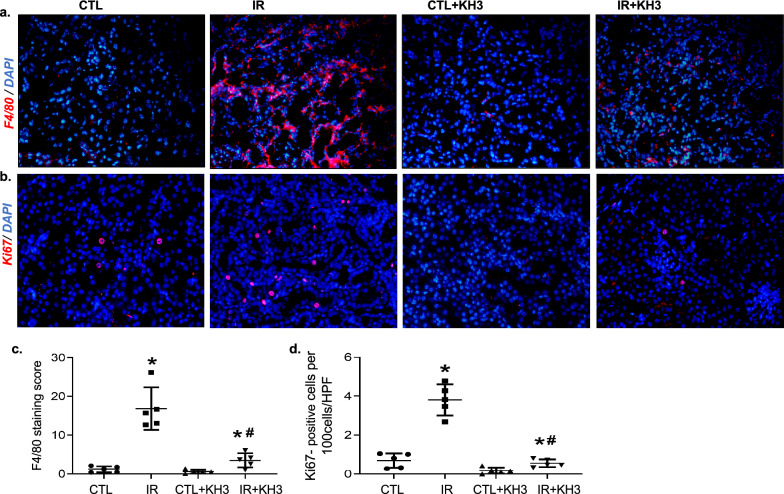
Treatment with KH3 reduces macrophage infiltration and tubular cell proliferation in the kidney after ischemia–reperfusion (IR) injury. **a**, **c** Kidney sections from untreated and KH3-treated mice (n = 5/each) following IR injury were stained with F4/80 (red) (**a**), Ki-67 (red) (**c**) and DAPI/DNA (blue) and analyzed using confocal microscopy. Magnification, ×200. The graphs summarized the results of F4/80^+^ (**b**) and Ki-67^+^ cells (**d**) quantified by image-J. *P < 0.05 vs. untreated contralateral kidney (CTL); ^#^P < 0.05 vs. untreated IR-injured kidney (IR)

After the confirmation of substantial elevation of inflammation and oxidative stress markers in IR-injured kidney tissue, we found the significant accretion of macrophages and inflammation in IR-injured kidneys which was evidenced by an increase in the absolute number of F4/80-positive macrophage cells in tubulointerstitial area of IR-injured kidney tissues. The number of F4/80-positive cells were scant in renal vessels of CTL kidneys. Nevertheless, F4/80-positive cells were markedly decreased with KH3 treatment in IR-injured kidneys (Fig. 6a, c). Ki-67 is a nuclear protein which assists as a cellular proliferation marker [30]. In our study, we found a significant upsurge of Ki-67-positive cells in tubulointerstitial area in IR-injured kidney tissues. In contrast, KH3-treated kidneys following IR injury had a much lower number of Ki-67-positive cells (Fig. 6b, d). In total, aforementioned data specify that inhibition of HuR with KH3 averts the expression and function of HuR-targeted molecules involved in IR injury-induced tubular inflammation, oxidative stress and abnormal cell proliferation thereby improving tubular fibrosis.

### Treatment with KH3 reduces apoptosis and peritubular capillary loss in the IR-injured kidney in mice

**Fig. 7:**
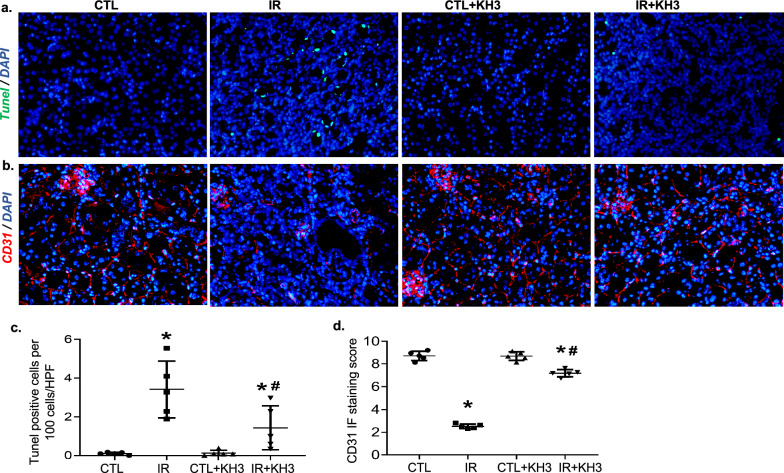
Treatment with KH3 reduces tubular cell apoptosis and peritubular capillary loss in the kidney after ischemia–reperfusion (IR) injury. **a**, **c** Kidney sections from untreated and KH3-treated mice (n = 5/each) following IR injury were stained with Tunel (green) (**a**), CD31 (red) (**c**) and DAPI/DNA (blue) and analyzed using confocal microscopy. Magnification, ×200. The graphs summarized the results of Tunel^+^ (**b**) and CD31^+^ cells (**d**) quantified by image-J. Magnification, ×200. *P < 0.05 vs. untreated contralateral kidney (CTL); ^#^P < 0.05 vs. untreated IR-injured kidney (IR)

Extensive apoptosis of renal tubular epithelial cells is also a pathological change of renal IR injury and was assessed by TUNEL assay in the present mouse model. As shown in Fig. 7a, a significant elevation in TUNEL+ cells, mainly in tubular cells (stained green) was observed in IR-injured kidney sections, compared with the CTL kidneys, which was markedly reduced when treated with KH3 (Fig. 7a, c).

Destruction of peritubular capillaries (PTC), known as rarefaction, is believed to be one of major damages and a key drive force of tubular fibrosis following IR injury [31, 32]. Here, we briefly evaluated the changes of PTC by immunofluorescent staining using anti-capillary endothelial cell marker, CD31 antibody. As shown in Fig. 7b, d, the microvasculature in the normal kidney cortex had normal density and architecture evident by normal density and distribution of CD31^+^ cell staining. Following IR injury, the number of PTC significantly decreased in the tubulointerstitial area, which was nearly reversed by KH3 treatment. Such additional effect of inhibition of HuR on apoptosis and peritubular capillary re-generation also explains the reduced fibrosis seen in KH3-treated mice with IR-induced chronic kidney injury.

### Changes of profibrotic pathways involved in inhibition of HuR in mice

**Fig. 8:**
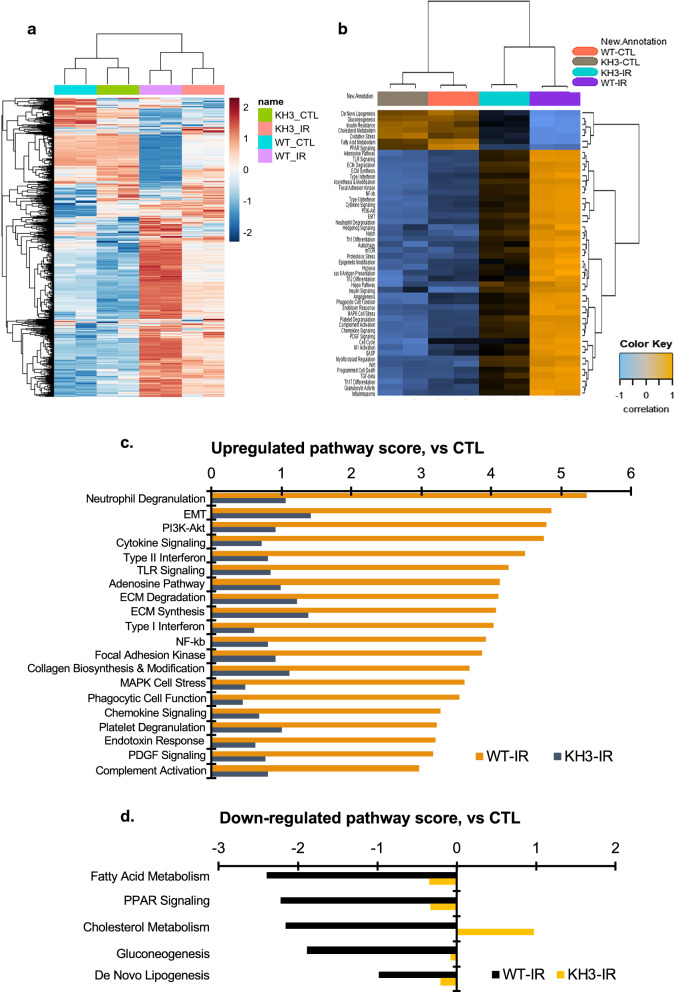
Heatmaps of differential mRNA expression and related pathway dysregulation scores for 760 molecules in the kidney after ischemia–reperfusion (IR) injury analyzed by the NanoString mouse nCounter Fibrosis Panel. **a** Differential mRNA expression map in IR-injured kidney between untreated (wild type, WT) and KH3-treated wild type mice(KH3) (n = 5/each group) compared to uninjured contralateral kidney (CTL), generated by using the limma software. **b** Dysregulation score map of 50 annotated profibrotic pathways in those kidneys generated by using nSlover™. **c** The top 20 upregulated profibrotic pathways and **d** Five down-regulated cellular energy metabolic pathways induced by IR injury, compared with the pathway scores in uninjured contralateral kidney (CTL) are remarkably ameliorated when KH3 was given

As shown in Fig. 8a, a heatmap of mRNA expression, differential mRNA expression (adjusted P < 0.05) in the IR-injured kidneys relative to their uninjured contralateral kidneys (CTL) (WT-IR vs WT-CTL) for 519 genes (367 genes were up-regulated and 152 genes were down-regulated) was clearly observed. The upregulated 367 genes include the molecules that we have observed in their mRNA expression or protein production individually described above and these genes encode for fibrotic markers; EMT markers; inflammatory factors and oxidative enzymes; growth factors and proliferation markers, and the molecules that we did not measure individually, for instance, caspase-4 (vs. CTL, 6.00-fold ↑, p = 0.002), and − 12 (3.94-fold ↑, p = 3.11E−06) and Bax (1.22-fold ↑, p = 0.0019) that regulate inflammation and apoptosis. However, all these increased molecules were attenuated in the KH3-treated and IR-injured kidneys of mice (KH3-IR vs WT-IR). In this model, downregulated genes were repeatedly observed in this study as our previous report [23], including SOD1 (superoxide dismutase 1) [33], Hmox1 (heme oxygenase 1) [34] and Txn1, 2 (thioredoxin 1, 2) [35] that belong to the antioxidant family and are associated with the enhanced oxidative stress occurred in the IR-injured kidney. In addition, mRNA levels of Vegf-a (vs. CTL, 0.66-fold ↓, p = 1.69E−05), Vegf-b (0.74-fold ↓, p = 0.0035) and their receptor Flt1 (0.71-fold ↓, p = 0.0018) were also reduced in the IR-injured kidneys, which may be related to IR-induced destruction of peritubular capillaries as observed in Fig. 8. However, such reduction in mRNA expression of antioxidants and modulators involved vasculature regeneration was largely ameliorated in the KH3-treated and IR-injured kidneys. In total, 247 of 367 upregulated genes were reduced (total 67.3%↓, with adjusted P < 0.05) and 123 of 152 downregulated gene were reversed to varying degrees (total 80.9%↑, with adjusted P < 0.05) in the IR-injured kidney treated with KH3 compared to non-treated (KH3-IR vs WT-IR). These molecules were involved in 50 ameliorated annotated profibrotic pathways (shown in Fig. 8b, a heatmap of pathway scores). Of them, the markedly upregulated 20 pathways seen in the IR-injured kidney, are shown in Fig. 8c, which commonly contribute to the development of renal fibrosis, but were significantly ameliorated in the KH3-treated and IR-injured kidney. Interestingly, this analysis further revealed that renal tubular cell energy metabolism (as shown in Fig. 8d), largely destroyed in the IR-injured kidney, was persevered in the KH3-treated and IR-injured kidney. Together, these results revealed that the majority of molecules that are involved in renal inflammation and fibrosis are inhibited directly or indirectly by HuR inhibitor.

### Treatment with KH3 abrogates the TGFß1/HuR feedback circuit in cultured HK-2 cells

TGFß1 was elevated in the IR-injured kidney, which was inhibited effectively by inhibition of HuR. Recently, a few studies have shown that TGFß1 also induces HuR cytoplasmic translocation to form a possible TGFß/HuR feedback loop thereby further enhancing HuR’s function [36]. It is well established that export of HuR to the cytoplasm is a key prerequisite for its stabilizing effects on the HuR-targeted mRNAs, although the mechanisms underlying HuR-nucleo-cytoplasmic trafficking are not completely understood yet. Here, we investigated if inhibition of HuR was able to abrogate a possible TGFß1/HuR feedback loop that further regulates the fibrogenic responses in cultured human renal tubular epithelial cells.

**Fig. 9:**
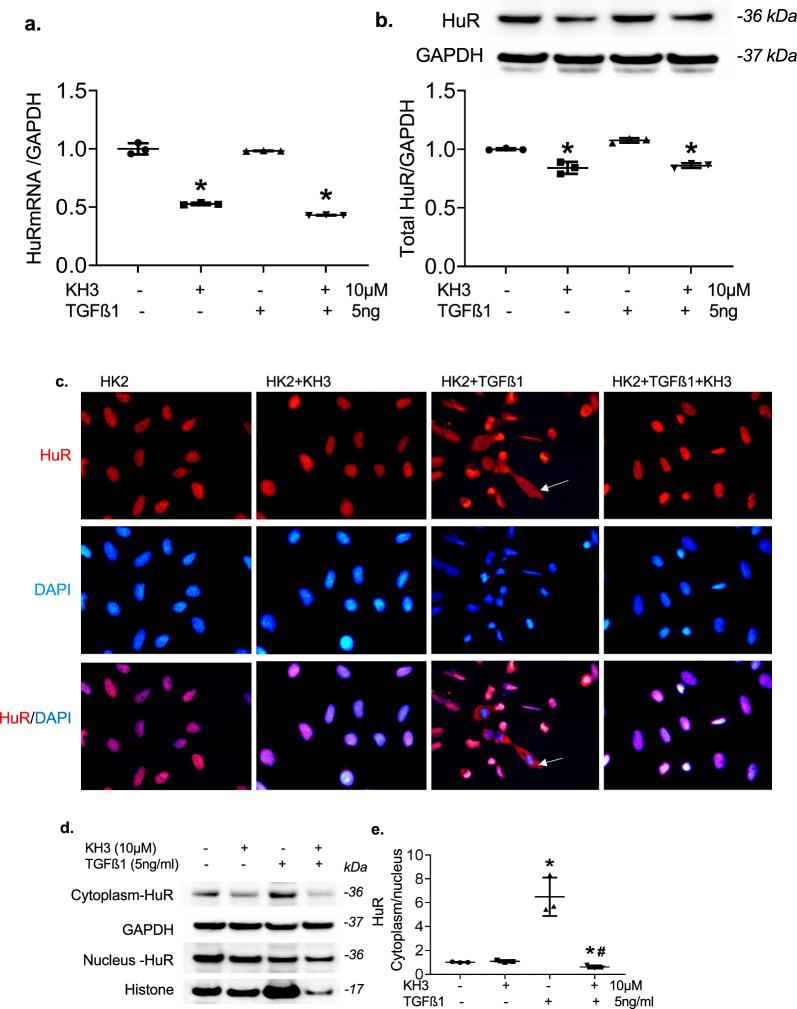
TGFß1 induces cytoplasmic translocation of HuR in cultured HK2 cells, which is inhibited by KH3. **a**, **b** mRNA expression (**a**) and total cellular protein production of HuR (**b**) in cultured HK2 cells. *P < 0.05, vs. cells without treatment. **c** Representative photomicrographs of cellular immunofluorescent staining for HuR (red) and DAPI (blue) (×400 magnification) from the un-treated HK2 cells (HK2), or HK2 cells treated with KH3 (HK2+KH3) or TGFß1 (KH2+TGFß1) alone or TGFß1 plus KH3 (HK2+TGFß1+KH3). Arrows indicate cytoplasmic staining of HuR. **d** Representative Western blots illustrating cytoplasmic and nucleus HuR and GAPDH and histone protein expression in untreated and treated HK2 cells. **e.** The graphs summarize the results of band density measurements for the ratio of cytoplasmic HuR over nucleus HuR. The ratio values are expressed relative to untreated cells, which was set at unity. *P < 0.05, vs. untreated cells; ^#^P < 0.05, vs. TGFß1 alone treated cells

**Fig. 10:**
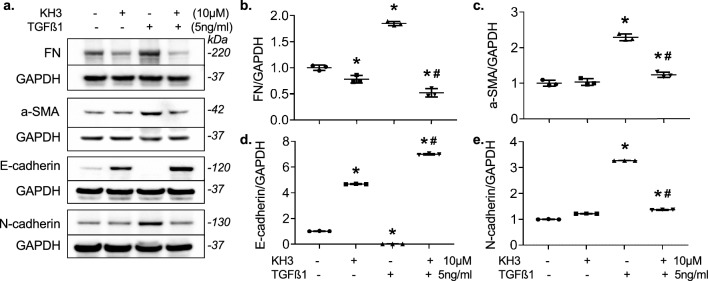
Treatment with KH3 abrogates TGFß1-induced renal tubular cell EMT in cultured HK2 cells. **a** Representative Western blots illustrating FN, a-SMA, E-cadherin, N-cadherin and GAPDH protein expression in untreated or treated HK2 cells. **b**–**e** The graphs summarize the results of band density measurements for FN (**b**), a-SMA (**c**), E-cadherin (**d**) and N-cadherin (**e**), respectively. The protein values are expressed relative to untreated cells, which was set at unity. *P < 0.05, vs. untreated cells; ^#^P < 0.05, vs. TGFß1 alone treated cells

**Fig. 11:**
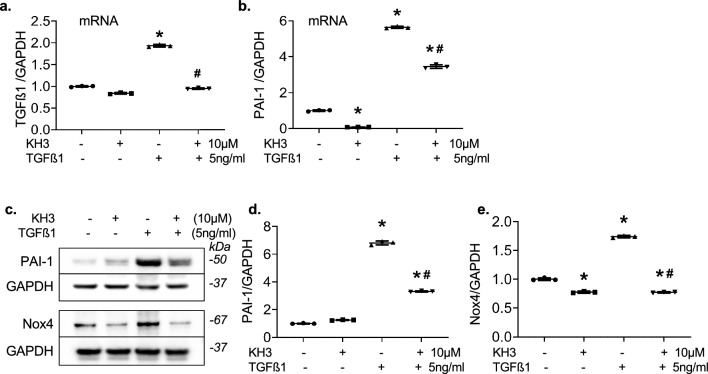
Treatment with KH3 inhibits TGFß1-induced cellular generation of TGFß1, PAI-1 and Nox4 in cultured HK2 cells. **a**, **b** mRNA expression of TGFß1 (**a**) and PAI-1 (**b**) in cultured HK2 cells. **c** Representative Western blots illustrating PAI-1, Nox4 and GAPDH protein expression in untreated or treated HK2 cells. **d**, **e** The graphs summarize the results of band density measurements for PAI-1 (**e**) and Nox4 (**e**), respectively. The protein values are expressed relative to untreated cells, which was set at unity. *P < 0.05, vs. untreated cells; ^#^P < 0.05, vs. TGFß1 alone treated cells

As shown in Fig. 9, TGFß1 stimulation did not change total cellular HuR at mRNA and protein levels in HK2 cells (Fig. 9a, b). However, administration of TGFß1 caused remarkable HuR cytoplasmic translocation as shown in Fig. 9c by cytoplasmic positive immunofluorescent staining for HuR and increased ratio of cytoplasmic over nuclear HuR protein levels determined by Western blot assay (Fig. 9d, e). Inhibition of HuR with KH3 not only reduced baseline HuR mRNA and protein expression but also blocked TGFß1-induced cytoplasmic translocation of HuR in cultured HK2 cells. Furthermore, inhibition of HuR with KH3 reversed TGFß1-induced overproduction of FN, a-SMA and N-cadherin and TGFß1-induced reduction of E-cadherin by HK2 cells, representing the tubular epithelial-to-mesenchymal/fibrotic phenotype transition (Fig. 10a–e). Moreover, inhibition of HuR with KH3 inhibited TGFß1-induced endogenous overexpression of TGFß1 and PAI-1 mRNAs and production of PAI-1 and Nox4 in cultured HK2 cells (Fig. 11a–e), which are involved in inflammation, EMT, matrix remodeling and fibrosis. Specially, KH3 alone is also able to inhibit FN but increase E-cadherin baseline expression, which may be resulted from KH3-induced decreases in baseline PAI-1 and Nox4 expression. Overall, these in vitro results revealed that inhibition of HuR with KH3 inhibits not only cellular TGFß1’s generation and profibrotic action but also the positive TGFß1/HuR feedback loop, which may effectively lead to reduction of renal tubulointerstitial fibrosis in vivo.

## Discussion

In the present study, we observed abnormal elevated tubular HuR abundance and nucleocytoplasmic transporting at the site of tubular injury besides glomeruli in kidney biopsies from patients with progressive CKD. Furthermore, we used the unilateral renal IR mouse model with a typical ischemia time [37] and observed that HuR was selectively upregulated at the site of IR-injured tubular and tubulointerstitial cells in mouse kidneys. A shift of HuR to the cytoplasm, where it exerts its role in mRNA stabilization, was also observed and increased there. This finding is consistent with our and others’ previous findings in diseased glomeruli and other kidney diseases, showing that local cellular HuR is aberrantly increased in kidney diseases in response to various forms of damage/injury [12, 16, 17, 38, 39], and the kidney transcriptome data that show mRNA expression levels of HuR in kidneys from CKD patients are much higher than those in healthy living donors (2.113-fold ↑, p = 0.006, www.nephroseq.org). Although the mechanism of HuR overexpression and enhanced cytoplasmic translocation has not been investigated thoroughly in kidney diseases, the concurrently elevated mRNA expression and function of profibrotic and proinflammatory factors observed in present and previous studies, as the putative targets of HuR, suggest that excessive upregulation of HuR is causally linked to the onset or progression of kidney fibrosis and might provide us with the better molecular target to prevent or reverse kidney fibrosis. Importantly, the HuR inhibitor, KH3, inhibits tubular HuR generation and action and markedly attenuates IR-induced tubulointerstitial injury and fibrosis, further confirming a pro-injury role of elevated HuR, as a key mediator, in kidney disease and the therapeutic potential of inhibition of HuR for inhibition of tubulointerstitial fibrosis and the progression of AKI to CKD.

In this study, we briefly screened the mRNA changes of HuR targets in KH3 treated IR-injured kidneys by using the NanoString mRNA expression, in order to understand the mechanism(s) of the KH3’s action. As expected, the putative targets of HuR involved in multiple pathways such as the inflammatory process, promotion of cell proliferation, EMT, matrix remodeling and tissue fibrosis that have been identified in cancer traits and CKD [28, 39] are also upregulated in IR-injured kidneys but markedly ameliorated when the interaction of HuR-mRNA of HuR targets is specifically inhibited by KH3. In addition, we further observed that TGFß1 induced the translocation of nuclear HuR into the cytoplasm in renal tubular epithelial cells as Dr. Bai et al. observed in fibroblasts [36]. Apparently, both TGFß1 and HuR form a positive feedback circuit to exacerbate the fibrogenic responses in renal tubular cells besides in fibroblasts, during the process of kidney injury. Treatment with KH3 decreased HuR expression in both mRNA and protein levels in vivo and in vitro in the present study, similar to our previous report [17]. HuR abundance is known to be auto-regulated by a positive-feedback loop involving HuR interaction with the 3’-UTR of its own mRNA [40]. KH3 is able to disrupt HuR for binding to HuR own 3′-UTR, thereby reducing HuR mRNA stability, abundance and nucleocytoplasmic transporting directly. Therapeutic effect of KH3 for IR-induced kidney injury and fibrosis may result from not only downregulation of the common pro-injury targets of HuR, but also downregulation of HuR own abundance, and abolishment of the TGFß1/HuR feedback loop.

Additionally, we observed that inhibition of HuR with KH3 ameliorated IR-induced tubular and tubulointerstitial cell apoptosis and peritubular capillary loss. IR damage of tubular cells is a complex process which involves apoptosis and regeneration at the same time. When the renal ischemia beyond a certain point such as 35 min that we used for the mouse model may cause irreversible kidney tubular injury and the transition of AKI to CKD. This time is named as “a point of no return” ischemia time for mice [21]. It is why we observed extensive inflammation and cell apoptosis at 2 weeks after IR injury, accompanied by overexpression of caspases 4 and 12 and Bax in the IR-injured mouse kidney. Both caspases 4 and 12 are localized mainly in the cellular endoplasmic reticulum (ER) and can be activated by all the proinflammatory agents and ER stress inducers and mediate ER stress-induced cell death in many cells [41, 42]. Bcl-2-associated X (Bax) is a proapoptotic member of the Bcl-2 family that governs mitochondrial outer membrane permeabilization to elicit apoptotic cell death [43]. Overexpression of caspases 4 and 12 and Bax is likely associated with increased apoptotic and necrotic cell death as observed in the IR-injured mouse kidney in this study. It is yet unknown whether caspases 4 and 12 and Bax are the direct HuR targets. However, that inhibition of HuR-mRNA interaction with KH3 downregulates targeted proinflammatory factors and ER-stress inducers should suppress caspase/Bax-mediated apoptotic cell death. This possible anti-apoptotic role of HuR inhibition is consistent with the role of HuR in promoting cell death observed in HeLa cells under persistent stress beyond repair such as a lethal stress [44], but conflicted with the role of HuR observed in renal tubule cells during energy depletion [15]. Many researchers have focused on understanding the proapoptotic or anti-apoptotic role of HuR. Indeed, HuR may function differently by targeting different targets during the two characteristic steps of the cell stress response. First, it participates in processes that activate pro-survival pathways to facilitate cell recovery. Then, if the stress becomes persistent, HuR promotes cell death via the caspase pathway [44, 45]. Our group also observed similar dual effects of HuR in apoptosis depending on the nature of stimuli applied in cancer cells (unpublished data). The proapoptotic effect of HuR revealed by the present study may result from IR-induced persistent lethal damage and stress to renal tubule cells. However, the cellular mechanism by which HuR promotes apoptosis in the progression of CKD needs to be further delineated.

Destruction of peritubular capillaries (PTC) is believed to be one of major damages and a key drive force of tubular fibrosis following IR injury due to exacerbated deficiency of oxygen/nutrient supply to capillary cells [31, 32]. To maintain the integrity of PTC is mainly related to several pro-angiogenetic modulators besides inhibition of capillary cell apoptosis after damage. We observed downregulated modulators involved vasculature regeneration such Vegf-a, Vegf-b and their receptor Flt1 in IR-injured kidneys but largely improved expression in KH3-treated IR-injured kidneys. The improved expression of Vegf and Vegf receptor likely contributes to ameliorated PTC regeneration in this model. However, this effect is conflicted with the role of HuR involved in renal angiogenesis in tumor environment and diabetic nephropathy, where HuR and AUF1 bind to Vegf-a-ARE promoting angiogenesis under both normoxic and hypoxic conditions [16, 46–48]. These discrepancies suggest that the overall effect of HuR-regulated vasculature regeneration in kidney tissue may vary in different situations. More efforts are needed to clarify the detailed role of HuR in the capillary regeneration of progressive CKD.

Energy depletion is a critical factor in the transition of renal AKI to CKD, even CKD to ESRD [49]. Although we did not measure renal ATP levels after IR injury in untreated mice, our brief observation using the NanoString gene expression in this model provides the first evidence that the enhanced HuR function may be involved in regulation of molecules that contribute to renal tubular cell mitochondrial energy metabolism such as mitochondrial fatty acid oxidation and lipotoxicity in kidney diseases. Inhibition of HuR likely prevents cellular energy disorder thereby ameliorating cellular apoptosis and kidney fibrosis. Such new possible paradigm of dysregulation of HuR-targeted and energy metabolism-related molecules has not been identified yet and deserves further investigation.

## Conclusions

In summary, accumulated evidence indicates that local cellular HuR is aberrantly increased in the kidney in response to various forms of damage/injury. The present study further demonstrates that HuR significantly increases at the site of tubular injury both in progressive CKD in patients and in IR-injured kidneys in mice, accompanied by upregulation of HuR targets that are involved in inflammation, profibrotic cytokines, oxidative stress, proliferation, apoptosis, tubular EMT process and matrix remodeling in renal tubulointerstitial fibrosis. KH3, a potent HuR inhibitor that targets the HuR-AREs interaction in the 3′-UTR of target mRNAs, significantly reduces the IR-induced tubular injury and fibrosis by a combination of mechanisms including inhibition of HuR its own abundance and function, downregulation of the generation and action of multiple HuR targets, and interruption of TGFß1/HuR feedback loop. The limitation of this study is that the influence of HuR inhibition on renal function was not able to be displayed directly in the present animal model without contralateral nephrectomy. The efficacy of HuR inhibition for IR-induced AKI such as at day 3 also needs to be further determined. Nonetheless, our results together with the therapeutic effect of KH3 for glomerulosclerosis we reported before [17] suggest that inhibition of HuR with KH3 holds great promise as a therapeutic agent for slowing/preventing the progression of kidney disease including glomerular and tubular fibrosis. Future clinical interventional trails are needed to confirm or refute this hypothesis.

## Supplementary Information

**Additional file 1: Table S1.** Antibody resource and working solution information (Part I). Antibody resource and working solution information (Part II). **Table S2.** Primers used for real time PCR.

## Data Availability

Please contact author for data requests. These data were presented in part at the annual kidney week of American Society of Nephrology; November 4 through 7, 2021.

## Abbreviations

CKD: Chronic kidney diseases
ESRD: End-stage renal disease
AKI: Acute kidney injury
EMT: Epithelial-to-mesenchymal transition
HuR: Human antigen R
ELAVL1: Embryonic lethal abnormal vision-like 1
IR: Ischemia/reperfusion
CTL: Contralateral
NC: Normal control
AREs: Adenine and uridine rich sequence elements
3′-UTR: 3′-Untranslated region
MTT: 3-(4,5-Dimethylthiazol-2-yl)-2,5-diphenyltetrazolium bromide
DMSO: Dimethyl sulfoxide
IgAN: IgA nephropathy
DN: Diabetic nephropathy
LN: Lupus nephritis
FSGS: Focal segmental glomerulosclerosis
TMA: Thrombotic microangiopathy
PAS: Periodic acid–Schiff
BUN: Blood urea nitrogen
Cr: Creatinine
ALT: Alanine aminotransaminase
TRI: Masson’s Trichrome
IF: Immunofluorescent staining
FITC: Fluorescein isothiocyanate
WGA: Wheat germ agglutinin
DAPI: 4′,6-Diamidino-2-phenylindole
a-SMA: α-Smooth muscle action
FN: Fibronectin
TGFß1: Transforming growth factor-beta1
PAI-1: Plasminogen Activator Inhibitor Type 1
NF-kB-p65: Nuclear factor κ-B p65 unite
Nox2: NADPH oxidase gp91^phox^
Nox4: NADPH Oxidase 4
ERK1/2: Extracellular signal-regulated protein kinase 1 and 2
GAPDH: Glyceraldehyde-3-phosphate dehydrogenase
RT-PCR: Reverse transcription-polymerase chain reaction
TUNEL: The enzyme terminal deoxynucleotidyl transferase-mediated dUTP nickend labeling
SD: Standard deviation
NGAL: Neutrophil gelatinase-associated lipocalin
KIM-1: Kidney injury molecule-1
Col-Iα1: Collagen type Iα1
Col-IIIα1: Collagen type III α1
MCP-1: Monocyte chemoattractant protein-1
PTC: Peritubular capillaries
SOD: Superoxide dismutase 1
Hmox1: Heme oxygenase 1
Txn1: Thioredoxin 1
Vegf-a/b: Vascular endothelial growth factor A/B
Flt1: Fms Related Receptor Tyrosine Kinase 1
ER: Endoplasmic reticulum
Bax: Bcl-2-associated X
ATP: Adenosine triphosphate
