# Supplementary material for: Targeting RNA-binding protein HuR to inhibit the progression of renal tubular fibrosis
Source: J Transl Med. 2023 Jun 30;21:428. doi: 10.1186/s12967-023-04298-x (PMC10311833; doi:10.1186/s12967-023-04298-x)
Supplement: Supplementary file 1 — Additional file 1: Table S1. Antibody resource and working solution information (Part I). Antibody resource and working solution information (Part II). Table S2. Primers used for real time PCR. [file 12967_2023_4298_MOESM1_ESM.pdf]

Additional file 1 for

Targeting RNA-binding protein HuR to inhibit the progression of renal tubular fibrosis

Zhimin Huang[1§], Simeng Liu[1§], Anna Tang[1], Xiaoqing Wu[2], Jeffrey Aube[3], Liang Xu[2], and Yufeng Huang[1*]. 1. Department of Internal Medicine, Division of Nephrology & Hypertension, University of Utah Health Science, Salt Lake City, UT, USA. 2. Department of Molecular Biosciences, University of Kansas, Lawrence, KS, USA. 3. Department of Chemical Biology and Medical Chemistry, Eshelman School of Pharmacy, University of North Carolina, Chapel Hill, NC, USA.

§ equally contributed to this paper.

* Address correspondence to: Dr. Yufeng Huang, Division of Nephrology & Hypertension, Department of Internal Medicine, University of Utah Health Science, Wintrobe Rm 403, 26 N Medical Dr., Salt Lake City, UT 84132, USA, Phone: 801-585-0581, Fax: 801-213-2563, Email: Yufeng.huang@hsc.utah.edu.

Table S1.

Antibody resource and working solution information (Part I)

1st antibody	Resource Company	Catalog number	Assay	Dilution fold
Mouse anti-HuR IgG	Santa Cruz Biotechnology Inc, Santa Cruz, CA, USA	sc-5261	IF	1:100
Alexa Fluor^{TM} 488-conjugated wheat germ agglutinin (WGA)	ThermoFisher Scientific, USA	W11261	IF	1:100 (10μg/ml)
FITC-conjugated-mouse anti-a smooth muscle actin (a-SMA) antibody	Sigma-Aldrich, St. Louis, MO, USA	F3777	IF	1:50
Rat anti-mouse CD31 IgG2a	BD Biosciences, San Jose, CA, USA	553370	IF	1:100
Rat anti-mouse F4/80 IgG2b (CL: A3-1)	Bio-Rad Laboratories, Inc. Hercules, CA, USA	MCA497R	IF	1:50
Rat anti-Ki-67 antibody (SolA15)	Invitrogen, Carlsbad, CA, USA	14-5698-82	IF	1:100
Recombinant Rabbit anti-HuR antibody	Abcam, Cambridge, U.K.	Ab200342	WB	1:1000
Mouse monoclonal anti-a-SMA antibody	Sigma-Aldrich, St. Louis, MO, USA	A5228	WB	1:2500
Rabbit anti-Human FN IgG	Sigma-Aldrich, St. Louis, MO, USA	F3648	WB	1:1000
Mouse anti-E-cadherin IgG2a	BD Biosciences, San Jose, CA, USA	610181	WB	1:1000
Mouse monoclonal anti-N-cadherin (H-2) IgG1	Santa Cruz Biotechnology Inc, Santa Cruz, CA, USA	sc-393933	WB	1:2000
Rabbit anti-Vimentin IgG	GenScript Biotech, Piscataway, NJ, USA	A01191	WB	1:500
Rabbit anti-TGFß1 IgG	Abcam, Cambridge, U.K.	Ab92486	WB	1:250
Mouse anti-PAI-1 IgG1	BD Biosciences, San Jose, CA, USA	612025	WB	1:1000
Mouse monoclonal anti-NF-kBp65 antibody	Cell Signaling Technology,	6956s	WB	1:500
Mouse anti-gp91[phox] (Nox2)IgG1	BD Biosciences, San Jose, CA, USA	611414	WB	1:500
Mouse anti-human $p47^{phox}$ IgG1	BD Biosciences, San Jose, CA, USA	610354	WB	1:1000
Rabbit anti-Nox4 IgG	Sigma-Aldrich, St. Louis, MO, USA	ABC459	WB	1:2000
Mouse monoclonal anti-p-ERK1/2 (E-4) IgG2a	Santa Cruz Biotechnology Inc, Santa Cruz, CA, USA	Sc-7383	WB	1:500
Rabbit-anti-total ERK1/2 IgG	Santa Cruz Biotechnology Inc, Santa Cruz, CA, USA	Sc-153	WB	1:1000
Goat anti-GAPDH antibody	GenScript Biotech, Piscataway, NJ, USA	A00191	WB	1:2500
Rabbit-anti-Histone H3	Abcam, Cambridge, U.K.	Ab1791	WB	1:1000

IF: immunofluorescent staining, WB: western blot assay.

Antibody resource and working solution information (Part II)

2nd antibody	Resource Company	Catalog number	Assay	Dilution fold
Alexa Fluor™ 594-conjugated goat anti-mouse IgG (H+L)	Invitrogen, Carlsbad, CA, USA	A-11005	IF	1:200
Cy™3-conjugated goat anti-rat IgG	Jackson ImmunoResearch Laboratories Inc., West Grove, PA, USA	112-165-143	IF	1:400
4', 6-diamidino-2-phenylindole (DAPI)-Fluoromount-G	SouthernBiotech, Birmingham, AI, USA	0100-20	IF	No dilution
HRP-conjugated donkey anti-rabbit IgG	Jackson ImmunoResearch Laboratories Inc.	711-035-152	WB	1:1000 or 1:2000
HRP-conjugated goat anti-mouse IgG	Jackson ImmunoResearch Laboratories Inc.	115-035-146	WB	1:2000
HRP-conjugated rabbit anti-goat IgG	Jackson ImmunoResearch Laboratories Inc.	305-035-003	WB	1:2000
HRP-conjugated goat anti-rabbit IgG	Jackson ImmunoResearch Laboratories Inc.	111-035-144	WB	1:2000

IF: immunofluorescent staining, WB: western blot assay.

Table S2.

Primers used for real time PCR

Gene	Primer	Sequence 5'-3'
Mouse NGAL	Forward<br>Reverse	ATGTCACCTCCATCCTGGTC<br>ACAGCTCCTTGGTTCTTCCA
Mouse KIM-1	Forward<br>Reverse	ACATATCGTGGATCACAACGAC<br>ACTGCTCTTCTGATAGGTGACA
Mouse Col-Ia1	Forward<br>Reverse	ACGTCCTGGTGAAGTTGGTC<br>CAGGGAAGCCTCTTTCTCCT
Mouse Col-IIIa1	Forward<br>Reverse	GCACAGCAGTCCAACGTAGA<br>TCTCCAAATGGGATCTCTGG
Mouse FN	Forward<br>Reverse	CCGTGGGATGTTTGAGACTT<br>GGCAAAAGAAAGCAGAGGTG
Mouse TGFß1	Forward<br>Reverse	TTGCTTCAGCTCCACAGAGA<br>TGGTTGTAGAGGGCAAGGAC
Mouse PAI-1	Forward<br>Reverse	GTAGCACAGGCACTGCAAAA<br>ATCACTTGCCCCATGAAGAG
Mouse NF-kBp65	Forward<br>Reverse	CTTCCTCAGCCATGGTACCTCT<br>CAAGTCTTCATCAGCATCAAACTG

Mouse MCP-1	Forward<br>Reverse	AGCACCAGCCAACTCTCACT<br>CGTTAACTGCATCTGGCTGA
Mouse Nox2	Forward<br>Reverse	CCGTATTGTGGGAGACTGGA<br>CTTGAGAATGGAGGCAAAGG
Mouse GAPDH	Forward<br>Reverse	ACCCAGAAGACTGTGGATGG<br>CACATTGGGGGTAGGAACAC
Human HuR	Forward<br>Reverse	GGTGACATCGGGAGAACGAA<br>GGCGAGCATACGACACCTTA
Human TGFß1	Forward<br>Reverse	CTAATGGTGGAAACCCACAACG<br>TATCGCCAGGAATTGTTGCTG
Human PAI-1	Forward<br>Reverse	AGTGGACTTTTCAGAGGTGGA<br>GCCGTTGAAGTAGAGGGCATT
Human GAPDH	Forward<br>Reverse	GAAGGTCGGAGTCAACGGATTT<br>CTTGACGGTGCCATGGAATTT
